# Critical evaluation of HPV16 gene copy number quantification by SYBR green PCR

**DOI:** 10.1186/1472-6750-8-57

**Published:** 2008-07-24

**Authors:** Ian Roberts, Grace Ng, Nicola Foster, Margaret Stanley, Michael T Herdman, Mark R Pett, Andrew Teschendorff, Nicholas Coleman

**Affiliations:** 1 MRC Cancer Cell Unit, Hutchison/MRC Research Centre, Hills Road, Cambridge. CB2 0XZ, UK; 2 Department of Pathology, Cambridge University, Tennis Court Road, Cambridge. CB2 1QP, UK; 3 Cancer Research UK, Li Ka Shing Centre, Cambridge Research Institute, University of Cambridge, CB2 0RE, UK

## Abstract

**Background:**

Human papilloma virus (HPV) load and physical status are considered useful parameters for clinical evaluation of cervical squamous cell neoplasia. However, the errors implicit in HPV gene quantification by PCR are not well documented. We have undertaken the first rigorous evaluation of the errors that can be expected when using SYBR green qPCR for quantification of HPV type 16 gene copy numbers. We assessed a modified method, in which external calibration curves were generated from a single construct containing HPV16 E2, HPV16 E6 and the host gene hydroxymethylbilane synthase in a 1:1:1 ratio.

**Results:**

When testing dilutions of mixed HPV/host DNA in replicate runs, we observed errors in quantifying E2 and E6 amplicons of 5–40%, with greatest error at the lowest DNA template concentration (3 ng/μl). Errors in determining viral copy numbers per diploid genome were 13–53%. Nevertheless, in cervical keratinocyte cell lines we observed reasonable agreement between viral loads determined by qPCR and Southern blotting. The mean E2/E6 ratio in episome-only cells was 1.04, but with a range of 0.76–1.32. In three integrant-only lines the mean E2/E6 ratios were 0.20, 0.72 and 2.61 (values confirmed by gene-specific Southern blotting). When E2/E6 ratios in fourteen HPV16-positive cervical carcinomas were analysed, conclusions regarding viral physical state could only be made in three cases, where the E2/E6 ratio was ≤ 0.06.

**Conclusion:**

Run-to-run variation in SYBR green qPCR produces unavoidable inaccuracies that should be allowed for when quantifying HPV gene copy number. While E6 copy numbers can be considered to provide a useable indication of viral loads, the E2/E6 ratio is of limited value. Previous studies may have overestimated the frequency of mixed episomal/integrant HPV infections.

## Background

Cervical carcinoma is the second most common malignancy affecting women [1]. Most cases are squamous cell carcinomas (SCCs), which develop from precursor squamous intraepithelial lesions (SILs). At present it is not possible to discriminate progressive from non progressive SIL, leading to over-treatment of large numbers of women, with attendant physical and psychological morbidity.

Persistent infection by high risk human papillomavirus (HR-HPV) represents the most significant risk factor in development of cervical carcinoma [2,3], with HPV16 being the virus type most commonly seen in SCC. In cervical malignancy HPV is usually integrated into the host genome. Integration is characterised by retention of the viral oncogenes E6 and E7, and by disruption or loss of expression of the viral transcriptional repressor E2, leading to deregulated production of E6 and E7 [4].

Various PCR based assays have been devised to measure HPV copy number and physical state in cell lines and clinical samples [5-13], with a view to deriving clinically or biologically useful information. Viral load is often determined from levels of genes present in episomes and integrants (i.e. E6 or E7), while physical state is inferred from the E2/E6 (or E2/E7) ratio. The latter approach is based on the notion that episome only infections would harbour an equivalent copy number of E2 and E6, giving an E2/E6 ratio of 1, while integrated virus with loss of E2 would give an E2/E6 ratio of 0, and a mixed episomal and integrant infections would give ratios between 0 and 1.

Both TaqMan and SYBR green strategies have been developed for HPV gene PCR, with the latter approach offering advantages of simplicity and low cost. Existing methods adjust for sample cellularity, generally determining HPV gene copy numbers per unit mass of genomic DNA (gDNA), per copy of an independent host calibrator gene, or with reference to a cell line [5-8,11-14]. These approaches are potentially liable to error, for example through inaccuracies in DNA concentration measurement, or from errors in handling and pipetting template DNAs for the construction of separate calibration curves. Small inaccuracies in quantification or dispensing of template DNA can translate to large over- or under-estimates of viral content, on account of the large dynamic range and sensitivity of real time PCR.

Despite these concerns, and the potential use of quantitative PCR (qPCR) data in clinical decision-making, remarkably few previous studies have undertaken detailed technical evaluation of the performance of PCR methods for assessing HPV load and physical state. The existing reports have typically been carried out using DNA mixtures or cell lines, but not both, and have only rarely been performed to a good standard [10,11]. Where quantitative data is available it generally suggests substantial inaccuracy. One study showed a ten fold difference in the absolute values for HPV quantification between two laboratories (attributable to differences in the calibration standards used) [10], while another determined a viral load in the cervical SCC cell line SiHa of 37 copies per cell [13], which is far in excess of the approximately three copies actually present [15,16].

As the literature contains no adequate evaluation of the errors inherent in SYBR green qPCR analysis of HPV gene copy number, the present study sought to rectify this omission. We undertook detailed assessment of a modified SYBR green based qPCR strategy for absolute quantification of the copy number of HPV16 E2 and E6 genes, compared to a host diploid genome. We produced a single clone, NA6, containing the amplicons HPV16 E2, HPV16 E6 and host gene hydroxymethylbilane synthase (HMBS; located at 11q23.3) in a 1:1:1 ratio, for use in generating external calibration curves. In principle, this system may offer advantages of simplicity and comparability. Firstly, all external calibration curves are generated from one template dilution series, rather than several series required with independent calibration constructs (for example, three would be required for measurement of E2, E6 and HMBS), thereby reducing the amount of manual handling needed. Secondly, the amount of host gDNA in each test sample can be quantified directly with reference to the HMBS standard curve, rather than relying on an estimation of gDNA concentration by spectrophotometry. Thirdly, a standardised calibration construct such as NA6 may reduce inter-laboratory variation in HPV gene quantification.

We have undertaken rigorous evaluation of the performance of HPV16 SYBR green qPCR and present data documenting the potential sources of error, even in the 'best case scenario' of cell line analysis. We first used mixtures of HPV16 DNA and host gDNA, then cervical keratinocyte cell lines in which the HPV16 copy number and physical state were determined by Southern blot and densitometry, and finally HPV16-positive SCC tissue samples. We demonstrate the error range that should be anticipated when quantifying HR-HPV gene copy number by SYBR green qPCR. We also show that E2/E6 ratios are of limited use in assessing HR-HPV physical state, being of value only for identifying the subset of integrant-containing cells in which the E2/E6 ratio is near zero.

## Methods

### Cell lines

The HPV16 positive cervical SCC cell lines SiHa, and CaSki and the HPV negative cervical SCC line C33A (all at high passage) were obtained from the American Type Culture Collection (ATCC). We also used early and late passages from one long-term culture series of the W12 cell line (W12 Series1; W12.Ser1). W12 was established from a cervical low grade SIL and in long term culture recapitulates cervical neoplastic progression genetically and phenotypically [17-20]. All cell lines were propagated in monolayer culture, as described previously [18,21] or by the ATCC.

### Genomic DNA extraction

Genomic DNA (gDNA) was prepared from pelleted cells by overnight digestion with proteinase K, brief phenol chloroform extraction, ethanol precipitation, and removal of contaminating RNA by RNAse A. The purified gDNA was quantified by spectrophotometry.

### Cloning of E2, E6 and HMBS amplicons into NA6

E2, E6 and HMBS amplicons were cloned separately, following PCR amplification, into pcr^®^2.1 vector using the original TA cloning system (Invitrogen). Briefly, fragments of the E2 hinge region (81 bp), and E6 gene (80 bp) were amplified from the pSP64-HPV16 construct (which contains the 7.9 kb full length HPV16 genome [20]), while a section of HMBS (118 bp) was amplified from peripheral blood lymphocyte gDNA. PCR was carried out on an Applied Biosystems 9700 using the AmpliTaq Gold system (Perkin Elmer, UK) and comprised 1 × Taq Buffer, 1.5 mM MgCl2, 200 μM dNTPs, 300 μM Primer Pairs, and 2 U of Taq polymerase. PCR primers and cycling conditions are listed in Table 1.

**Table 1:** Primers used to generate NA6 and for qPCR.

**Primers**	**Sequence (5' to 3')**	**Product length (bp)**	**Reference**
**E2 F**	AAC GAA GTA TCC TCT CCT GAA ATT ATT AG	81	[13]
**E2 R**	CCA AGG CGA CGG CTT TG		
**E6 F**	GAG AAC TGC AAT GTT TCA GGA CC	80	[13]
**E6 R**	TGT ATA GTT GTT TGC AGC TCT GTG C		
**HMBS F**	GCC TGC AGT TTG AAA TCA GTG	118	[32]
**HMBS R**	CGG GAC GGG CTT TAG CTA		

*F *refers to forward primers and *R *to reverse primers.

**Figure 1:**
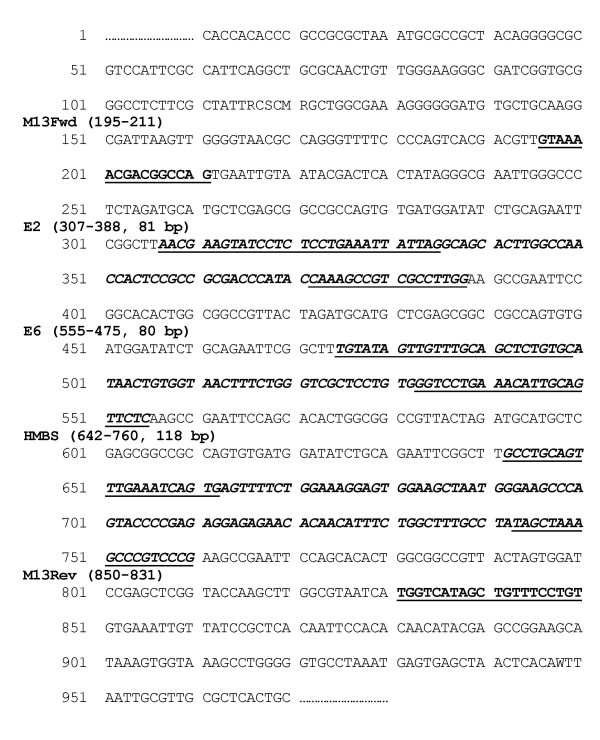
**Sequence of NA6 clone insert demonstrates that E2, E6 and HMBS amplicons are present in equimolar amounts**. The M13 forward primer of pcr2.1 TA vector is shown at 195 bp in bold, underlined. E2, E6 and HMBS amplicons are shown in bold italics, with primer binding sites underlined. The M13 reverse primer is at 850 bp, bold and underlined.

In order to construct a single clone containing each amplicon in a 1:1:1 ratio, the following cloning strategy was adopted. The E2 and E6 clones were linearised with XbaI, and the products ligated with T4 DNA ligase. Using the ligated product as template, PCR with E2 forward and E6 reverse primers generated a single E2-E6 product for subsequent TA cloning. Following selection, a single E2-E6 clone and a separate HMBS clone were digested with HindIII, and similarly ligated together. PCR with E2 forward and HMBS reverse primers generated a single product that following TA cloning, selection and sequencing, was shown to contain a single copy of the E2, E6 and HMBS amplicons (Figure 1). The final clone, named NA6, is 4,382 bp and has a molecular weight of 2.892 × 10^6 ^Da.

### Preparation of NA6 calibration curves

Stock NA6 was prepared at a concentration of 100 μg/ml. Ten fold serial dilutions were undertaken to produce a titration series representing, 1 ng/μl, 100 pg/μl, 10 pg/μl, 1 pg/μl, 100 fg/μl, 10 fg/μl and 1 fg/μl of NA6. From this, calibration curves for E2, E6 and HMBS were produced in quadruplicate. For each of the four qPCR runs, each dilution point was assayed in triplicate. qPCR was undertaken using 2 μl of template DNA in a 25 μl PCR. For the most concentrated point within the dilution series (1 ng/μl), 2 ng of NA6 equates to 4.176 × 10^8 ^copies of each amplicon. For the most dilute point, 2 fg equates to 4.176 × 10^2 ^copies. Additional file 1, 'NA6 Standard Curves' worksheet, presents the raw data used in calibration curve construction.

### SYBR Green qPCR

All qPCR was carried out using an Opticon I thermal cycler (MJ Research) with SYBR Green JumpStart qPCR Kits (Sigma, UK). Reactions comprised 1× SYBR Green mix, 500 nM primer pairs, and 2 μl of template. In initial optimisation experiments we investigated the effects of primer concentration on crossing point (Cp) determination. We found that a primer concentration of 500 nM was optimal, and that a reduction to 300 nM resulted in PCR artefacts at low template concentrations. It was found that a template volume of 2 μl could be dispensed more accurately and reproducibly than 1 μl volumes (data not shown).

The following cycling conditions were employed: initial denaturation 94°C 2 min, followed by 40 cycles of 94°C 20 seconds, 58°C 20 seconds, 72°C 20 seconds, 76°C 15 seconds, plate read. A final extension of 72°C 10 minutes, and melting curve of 65°C to 90°C, 1°C/second transition were incorporated. Opticon raw data was exported to Microsoft Excel for analysis.

### Data analysis

Absolute quantification strategies require that the fluorescence threshold used to derive the calibration curve is also applied to the sample data. Hence it may be anticipated that little effect would be experienced by varying the fluorescence threshold (Ft), provided that the Ft passes through the centre of the log transformed reaction curve data. However, we found that gross changes in absolute quantification were seen for relatively small changes in Ft following theoretical evaluations (data not shown). To avoid issues related to placement of Ft values, an automated derivation of optimum Ft assignment was implemented. Fluorescence thresholds were calculated for each of the four calibration curves according to the cycle before second derivative maxima method, and then averaged during the process of producing the calibration curve. The E2, E6 and HMBS Ft values were then applied to all sample data in order to calculate crossing points.

Calibration curves for E2, E6 and HMBS were constructed by plotting crossing points (Cp) versus the log of template copy number. For copy number determination in test samples, the fluorescence threshold (Ft), primer efficiency (E) and numbers of molecules at fluorescence threshold (Nt) were taken to be constants and were determined as the means of the four standard curve replicates for each amplicon. Hence, for an unknown sample, number of copies is given by equation 1.

(1) $$Copies=\frac{Nt}{{\left(1+E\right)}^{\text{Cp}}}$$

Calibration curve primer efficiencies were determined by equation 2, and numbers of molecules at threshold by equation 3.

(2) $$E=\left[10^{\frac{-1}{\text{slope}}}\right]-1$$

(3) *Nt *= 10^intercept^

The coefficient of variation between data obtained in replicate calibration curves represented the ratios of standard deviations over the mean, multiplied by 100%.

Gene copy numbers in test samples were obtained by comparing the Cp value to those in the relevant external calibration curve. A Microsoft Excel template was prepared to calculate viral loads, and E2/E6 ratios (see Additional file 1). For all samples, viral load per diploid genome was determined by dividing E2 and E6 copy numbers by half the HMBS copy number. In addition, for the cell lines, the viral load per cell was derived using the known ploidy status.

### Southern hybridisation

In order to validate the qPCR method, comparisons were made with HPV16 copy numbers determined in cervical keratinocyte cell lines by Southern blotting. 5 μg of gDNA was digested at 37°C overnight with either BamH1, PstI or HindIII, and electrophoresed on a 0.8% agarose gel. Agarose gels were depurinated in 0.25 M HCl, denatured in 0.5 M NaOH/1.5 M NaCl and neutralised in 0.5 M tris HCl/3 M NaCl before transfer to Hybond N membrane. UV cross-linked membranes were prehybridised in 20 ml of hybridisation buffer for 4 hrs at 65°C prior to introduction of ^32^P-dCTP labelled probe. Probes were generated from the previously described E2 and E6 amplicons (81 and 80 bp respectively) cloned into pcr2.1 TA cloning vector, or from full length HPV16 (7.9 kbp) excised from pSP64-HPV16 using BamH1, and labelled using RediPrime labelling kit. Hybridisation was for 16 hr, followed by stringency washing of membranes in 2 × SSC/0.1% SDS and 0.1 × SSC/0.1% SDS twice for 15 minutes, and then autoradiography.

### Clinical samples

Cervical squamous cell carcinoma samples were kindly provided by Dr Geetashree Mukherjee from the archives of the Kidwai Memorial Institute of Oncology, Bangalore, India. All tissue samples were obtained with informed consent, anonymised, and used with approval from the Kidwai Local Research Ethics Committee (reference: PER/CAB-I/D-I/13/01).

gDNA was prepared from 14 snap frozen HPV16-positive cervical SCC samples, as described elsewhere [21]. The mean total yields were insufficient for Southern blotting to be performed to provide comparative data (data not shown). Additional file 1, 'Assessment of Clinical Samples' worksheet, presents the raw data from the qPCR analysis of the clinical sample viral load and E2/E6 ratios.

## Results

### Validation of NA6 standard curves using mixtures of virus and host DNA

In generating the NA6 construct and preparing the calibration curves (see Materials and Methods), PCR primers were chosen to give amplicons of similar length (Table 1). The E2 primers, amplifying bp 3,361–3,442 of the HPV16 genome (GenBank accession AF125673), were located in the part of the E2 open reading frame (ORF) that is most often deleted on HPV16 integration [5]. The E6 primers were located in the E6 ORF (bp 94–174), while the HMBS primers spanned an exon-intron border. After cloning, the 1:1:1 relationship of the E2, E6 and HMBS amplicons in NA6 were confirmed by sequence analysis (Figure 1).

**Figure 2:**
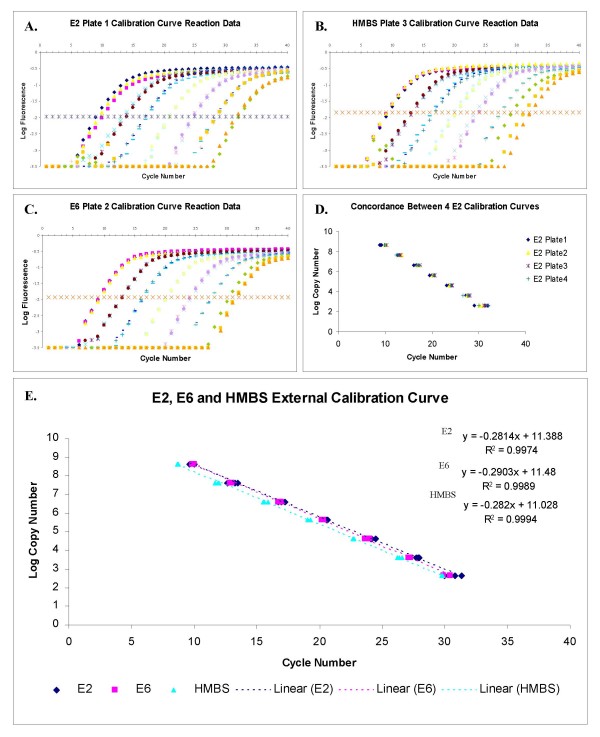
**Generation of mean NA6 calibration curve for E2, E6 amd HMBS quantification**. Panels A – C show representative data from the four replicate PCR runs using serially diluted NA6 as template (2 ng – 2 fg), from which the mean calibration curve for viral quantification was generated. The different curves correspond to different starting amounts of template. The fluorescence thresholds are indicated by the lines of crosses running horizontally. Panel A shows E2 log transformed data from replicate 1 qPCR run, panel B shows HMBS log transformed data from replicate 3 qPCR run, panel C shows E6 log transformed data from replicate 2 qPCR run. Panel D demonstrates the tight correlation between the data points generated for the E2 amplicon at each template concentration in the four replicate runs. Similar findings were made for the E6 and HMBS amplicons. Panel E shows the mean calibration curves for E2, E6 and HMBS used for viral gene copy number quantification, together with the respective line equations.

The SYBR green qPCR method used was developed following numerous optimisation experiments to determine ideal primer concentration, template gDNA concentration and cycling parameters (see Materials and Methods). We analysed melting curves of reaction products to verify specificity of primer binding and thereby circumvent any issues of non-specific SYBR green fluorescence. Following four replicate qPCR runs, seven point external calibration curves were generated for E2, E6 and HMBS. Figure 2A – C shows representative qPCR data used to generate standard curves for E2 (Fig 2A; from replicate 1 of 4), E6 (Fig 2B; from replicate 2 of 4), and HMBS (Fig 2C; from replicate 3 of 4). Quantification of each gene was linear over six logs, with no evidence to suggest competition between the PCR targets in the NA6 construct.

Tight correlations were observed between the values obtained for each amplicon in the replicate qPCR runs, as illustrated for the E2 amplicon in Figure 2D (R^2 ^= 0.996). For each amplicon, the mean Cp for each titration point 1 ng/μl to 1 fg/μl was plotted to generate the final calibration curves, which are shown in Figure 2E, together with their respective line equations and correlation values.

Despite the apparent concordance between replicate runs, when we calculated viral gene copy number using calibration curves generated from individual qPCR runs, we observed inter-assay differences in the copy number values generated. Each of the four replicate calibration curves (Replicate 1 to Replicate 4) for E2, E6 and HMBS amplicons, as well as the final mean calibration curve derived from the quadruplicate runs, were used to calculate gene copy number according to a series of theoretical crossing points (Cp; the cycle number where the fluorescence threshold was crossed), ranging from a Cp of 10 to 30 (Table 2). The inter-run coefficients of variation in quantification of gene copy number at each crossing point ranged from approximately 4% to 31% (see penultimate row of each section of Table 2), which is in agreement with previously published findings [5,10,11]. There was no apparent trend according to crossing point, with as much variation at low Cp values as at high Cp values (Table 2), indicating that, in contrast to other methods [10,11], template concentration did not affect inter-assay variation. Nevertheless, this data indicates that there is unavoidable run to run variation in HPV qPCR, reflecting for example, the consequences of repeated manual handling in replicate runs. We therefore suggest that the generation of a reliable standard curve for HPV gene quantification requires averaging of replicate calibration runs, rather than depending on a single run.

**Table 2:** Effect of standard curve selection on E6, E2 and HMBS gene copy number calculations.

**E6**	**Correlation Coefficient (R^**2**^)**	**Primer Efficiency (E)**	**Number of molecules at threshold (Nt)**	**E6 gene copy numbers over a range of theoretical crossing points**				
	
				**10**	**15**	**20**	**25**	**30**
**Final calibration curve for E6**	**0.9963**	**95.01%**	**3.3806E+11**	**4.25E+08**	**1.51E+07**	**5.34E+05**	**1.89E+04**	**6.72E+02**

**Replicate 1**	0.9988	87.79%	1.43162E+11	2.62E+08	1.12E+07	4.81E+05	2.06E+04	8.82E+02
**Replicate 2**	0.9909	101.86%	5.85431E+11	5.21E+08	1.56E+07	4.64E+05	1.39E+04	4.13E+02
**Replicate 3**	0.9982	96.56%	3.68007E+11	4.28E+08	1.46E+07	4.97E+05	1.69E+04	5.77E+02
**Replicate 4**	0.9973	93.84%	2.55641E+11	3.41E+08	1.25E+07	4.56E+05	1.67E+04	6.09E+02

			**Coefficient of variation of replicates**	28.72%	14.58%	3.83%	16.27%	31.32%
			**Mean copy number of replicates**	3.88E+08	1.35E+07	4.74E+05	1.70E+04	6.20E+02
								
**E2**	**Correlation Coefficient (R^**2**^)**	**Primer Efficiency (E)**	**Number of molecules at threshold (Nt)**	**E2 gene copy numbers over a range of theoretical crossing points**				
	
				**10**	**15**	**20**	**25**	**30**

**Final calibration curve for E2**	**0.9945**	**90.86%**	**2.42762E+11**	**3.79E+08**	**1.49E+07**	**5.90E+05**	**2.33E+04**	**9.02E+02**

**Replicate 1**	0.9887	88.55%	1.92376E+11	3.39E+08	1.42E+07	5.96E+05	2.50E+04	0.9887
**Replicate 2**	0.9945	93.10%	2.8539E+11	3.96E+08	1.48E+07	5.49E+05	2.05E+04	0.9945
**Replicate 3**	0.9973	92.70%	3.11657E+11	4.41E+08	1.66E+07	6.25E+05	2.35E+04	0.9973
**Replicate 4**	0.9976	89.07%	1.81623E+11	3.11E+08	1.29E+07	5.33E+05	2.21E+04	0.9976

			**Coefficient of variation of replicates**	15.69%	10.60%	7.36%	8.57%	13.06%
			**Mean copy number of replicates**	3.72E+08	1.46E+07	5.76E+05	2.28E+04	9.02E+02
								
**HMBS**	**Correlation Coefficient (R^**2**^)**	**Primer Efficiency (E)**	**Number of molecules at threshold (Nt)**	**HMBS gene copy numbers over a range of theoretical crossing points**				
	
				**10**	**15**	**20**	**25**	**30**

**Final calibration curve for HMBS**	**0.9970**	**91.05%**	**2.28115E+11**	**3.52E+08**	**1.38E+07**	**5.44E+05**	**2.14E+04**	**8.40E+02**

**Replicate 1**	0.9951	89.10%	1.333E+11	2.28E+08	9.43E+06	3.90E+05	1.61E+04	6.67E+02
**Replicate 2**	0.9985	95.39%	3.52583E+11	4.35E+08	1.53E+07	5.36E+05	1.88E+04	6.60E+02
**Replicate 3**	0.9990	90.43%	2.4809E+11	3.96E+08	1.58E+07	6.31E+05	2.52E+04	1.01E+03
**Replicate 4**	0.9953	89.26%	1.78485E+11	3.03E+08	1.25E+07	5.13E+05	2.11E+04	8.71E+02

			**Coefficient of variation of replicates**	27.36%	22.14%	19.17%	18.93%	20.99%
			**Mean copy number of replicates**	3.40E+08	1.32E+07	5.18E+05	2.03E+04	8.01E+02

In the main part of each section the rows represent the values for the final calibration curve and for the four replicate qPCR runs of the 7 point NA6 titration series, from which the final curve was generated. The right-hand five columns give the gene copy numbers determined from each curve at theoretical crossing points ranging from 10 to 30. In the bottom part of each section the numbers represent the coefficient of variation in mean copy number determined between the four replicate qPCR runs, together with the mean copy number determined from replicates 1 – 4 at each Cp.

We next assessed the ability of our absolute qPCR strategy accurately to quantify viral E2 and E6 copy number at a broad range of concentrations within a background of human DNA. The raw data from this work is presented in Additional file 1 'Accuracy Test' worksheet. Test DNA stock was generated by mixing 50 ng of human gDNA with 10 pg of the plasmid pSP64-HPV16 (which contains full-length HPV16 [20]) per microliter. The mixture was then used to produce a three point serial dilution (Table 3). The starting population theoretically represented 760,000 E2 and E6 amplicons, and 28,571 HMBS amplicons per 2 μl of NA6 template (the volume of template loaded in each PCR reaction), which equated to a load of 53 HPV16 copies per diploid genome.

**Table 3:** Dynamic range of E2, E6 and HMBS quantification by qPCR.

**Template DNA per μl**	**Expected quantification in 2 μl**					**Observed quantification with percent change over expected**							
		
	**E2**	**E6**	**HMBS**	**E2/E6**	**E6 per diploid genome**	**E2**		**E6**		**HMBS**		**E2/E6**	**E6 per diploid genome**
										
51 ng gDNA 10 pg pSP64 HPV16	760,000	760,000	28,571	1	53	893,063	**+17.5%**	616,115	**-18.9%**	49,816	**+74.4%**	1.4	25
11 ng gDNA 2.16 pg pSP64 HPV16	163,922	163,922	6,163	1	53	208,404	**+27.1%**	172,423	**+5.2%**	7,437	**+20.7%**	1.2	46
3 ng gDNA 60 fg pSP64 HPV16	44,665	44,665	1,679	1	53	26,956	**-39.7%**	40,597	**-9.1%**	1,047	**+37.6%**	0.7	78

Peripheral blood lymphocyte gDNA was spiked with a known quantity of HPV16 DNA (in the pSP64-HPV16 plasmid). The template DNA was serially diluted, and absolute amounts were measured by spectrophotometry. The template DNA values are given in the first column of the Table. The anticipated gene copy numbers for E2, E6 and HMBS were derived, and are shown in the 'Expected quantification' section. qPCR of the serially diluted samples was performed, and the observed gene copy numbers are reported in the 'Observed quantification' section.

DNA concentrations of each titration point were determined by spectrophotometry, measuring each sample three times. The mean values so determined were 51 ng/μl, 11 ng/μl and 3 ng/μl (Table 3) and these were used to calculate the anticipated amounts of the amplicons of E2, E6 and HMBS in the 2 μl of template assayed, for comparison with the values determined by qPCR.

Table 3 shows the mean data for each test sample (obtained from three replicate qPCR runs), compared with the predicted amounts of amplicon. Moderate variation (17.5–39.7% for E2 and 5.2–18.9% for E6) was seen at each concentration point; with greatest overall error at the lowest test sample concentration (3 ng/μl of gDNA; representing about 1,600 diploid cells per 2 μl of sample), where the limits of accuracy of qPCR at low template concentrations had presumably been exceeded.

When we determined viral gene copy number per diploid genome, we obtained figures of approximately 25 E6 copies per diploid genome in the 51 ng/μl sample; 46 E6 copies per diploid genome in the 11 ng/μl sample; and 78 E6 copies per diploid genome in the 3 ng/μl sample, compared to the anticipated 53 copies per diploid genome (Table 3). These figures represent errors of 53%, 13%, and 47% respectively. The error is not surprising given the run to run variation we observed in the qPCR optimisation reactions, plus the fact that the experiments we performed required serial dilution of test samples and measurement of DNA concentrations by spectrophotometry, of which represent potential sources of error. This error in absolute quantification of HPV gene copy number by qPCR should be anticipated in future studies.

### Estimation of viral load and physical status in HPV16 infected cell lines

We next assessed the performance of NA6-based absolute SYBR green qPCR in cervical keratinocyte cell lines in which the copy number and physical state of HPV16 were determined using Southern blotting and densitometry. Raw data from this assessment is presented in Additional file 1 'Assessment of Cell Lines' worksheet.

**Figure 3:**
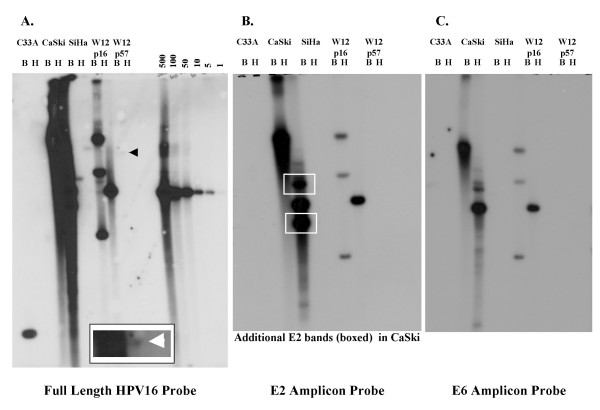
**Southern analysis of HPV16 copy number and physical state in the cervical keratinocyte cell lines used to validate the NA6 qPCR method**. The three panels represent blots probed with full length HPV16 (A), the HPV16 E2 amplicon (B) and the HPV16 E6 amplicon (C). The lanes marked B are BamH1 digests, while the lanes marked H are Hind III digests. The right hand lanes in Panel A show copy number controls for estimates of viral load, ranging from 500 to 1 copies of full length HPV16 per 5 μg of normal peripheral blood lymphocyte gDNA. C33A is HPV16 negative, CaSki contains integrated HPV16 at high copy, SiHa contains integrated HPV16 at low copy, W12.Ser1p16 contains episomal HPV16, and W12.Ser1p57 contains a single HPV16 integrant (arrowed). The inset at the bottom of Panel A shows the faint signal produced by the HPV16 integrant in W12.Ser1p57 following prolonged (72 hr) exposure. For CaSki cells, the signal produced with the E2 probe (Panel B) is considerably greater than that with the E6 probe (Panel C) and additional E2 bands are present (boxed in Panel B), indicative of multiple integration sites.

We undertook Southern hybridisation of BamH1 and HindIII digested gDNA from the cell lines C33A, W12.Ser1p16, W12.Ser1p57, CaSki, and SiHa (Figure 3, panels A-C). We used three different probes: full length HPV16 (7.9 Kbp), and the previously cloned E2 and E6 amplicons (81 and 80 bp respectively). For each probe, target sequence load was determined by autoradiographic comparison with copy number controls generated from full length HPV16, excised from the pSP64-HPV16 construct, and used at a range of 500 to 1 copy per 5 μg of host gDNA (right-hand lanes in Figure 3A). Viral loads were determined per cell, taking ploidy into account. The data was compared with viral loads determined by qPCR assessment of HPV16 E2 or E6 amplicon copies per diploid genome (Table 4). The HPV negative cervical SCC cell line C33A showed no signal by Southern blot and the viral gene copy number values determined by qPCR were also zero.

**Table 4:** Comparison of HPV16 viral load and physical state determined by Southern hybridisation with NA6 qPCR analysis of HPV16 E2 and E6 copy numbers in cervical keratinocyte cell lines.

**Cell line**	**Ploidy**	**Southern analysis Copy numbers per cell**	**NA6 qPCR analysis**			
			
			**E2 copies per diploid genome**	**E6 copies per diploid genome**	**Viral load per cell**	**Ratio of E2/E6 (range)**
**C33A**	Near triploid	HPV16 negative	0	0	0	0.00
**W12.Ser1p16**	Diploid	Episomal (approx 100 copies)	148	143	143	1.04 (0.76–1.32)
**W12.Ser1p57**	Tetraploid	Integrated (approx 1 copy)	0.2	0.3	1	0.72 (0.57–3.82)
**SiHa**	Triploid	Integrated (2–3 copies)	0.7	3.4	5	0.20 (0.11–0.24)
**CaSki**	Tetraploid	Integrated (>1000 copies)	2,272	869	1,738	2.61 (1.37–3.46)

Values less than 1 were rounded to the nearest whole number when calculating viral load per cell. E2/E6 ratio values were calculated from the qPCR readings for E2 and E6 and rounded to the nearest two decimal places.

Using full length HPV16 as probe, W12.Ser1p16 (known to be diploid [18]) was found to contain approximately 100 HPV16 episomes per cell, with no detectable integrants. qPCR indicated 143 E6 copies per diploid genome and 148 E2 copies per diploid genome. This represented a viral load of 143 copies per cell. The mean E2/E6 ratio determined from the absolute qPCR data was 1.04, as would be predicted in cells containing episomes only, although the range in triplicate qPCR runs was 0.76 to 1.32.

By Southern blotting using full length HPV16, W12.Ser1p57 (known to be near tetraploid [18]) was episome free and only a faint signal indicative of a single viral integrant was seen after 72 hrs exposure (Figure 3A). There were no detectable signals using the E2 or E6 amplicons as probe, even after 8 days of exposure, reflecting the predictably limited sensitivity of Southern blotting using 80 or 81 bp probes. By qPCR we observed a relatively wide range of copy number values in W12.Ser1p57, consistent with increased error at low sample concentrations (see Table 3). The mean copy number values determined from the absolute quantification data were 0.2 E2 copies and 0.3 E6 copies per diploid genome, indicating a viral load of approximately one copy per tetraploid cell (after rounding up). The mean E2/E6 ratio determined from the qPCR data was 0.72, with a range of 0.57 to 3.82. The presence of amplifiable E2 in W12.Ser1p57 is not surprising, as we previously determined retention of the hinge region in the HPV16 integrant in these cells [18].

SiHa cervical SCC cells (which are near triploid [22]) were shown by Southern blotting using full length HPV16 to be episome free and to contain approximately 3 integrants per cell, in keeping with previous reports [16,23]. Southern hybridisation using the E6 probe also suggested a viral load of approximately 3 copies per cell, whereas the E2 probe did not detect a signal. By qPCR SiHa was calculated to contain 3.4 copies of E6 per diploid genome, indicating a viral load of 5 copies per triploid cell (after rounding down). By qPCR, SiHa was also calculated to contain 0.7 copies of E2 per diploid genome, indicating error at the limits of qPCR performance, as the E2 hinge region is not retained in SiHa [15]. The mean E2/E6 ratio for SiHa was 0.20, with a range of 0.11 to 0.24.

CaSki cervical SCC cells (which are near tetraploid [22]) were shown by Southern blotting using full length HPV16 probe to be episome free and to contain more than 1,000 integrated copies per cell. By qPCR, CaSki was determined to carry approximately 2,272 E2 copies, and 869 E6 copies per diploid genome, the latter equating to a viral load of 1,738 E6 copies per tetraploid cell. The mean E2/E6 ratio for CaSki was 2.61, with a range of 1.37 to 3.46. Southern hybridisation using the E2 and E6 probes confirmed the presence of greater copy numbers of E2 than E6 in CaSki (Figure 3; compare panels B and C), and 1-dimensional analysis of the autoradiogram gave an E2/E6 ratio of approximately 2.

The data from the detailed cell line analysis confirms that errors in SYBR green qPCR-based HPV16 gene quantification should be allowed for, especially at low copy number. Nevertheless, the viral loads determined from mean E6 copy numbers were reasonably close to the values shown by Southern blotting and can be considered to provide a useable indication of actual loads. The E2/E6 ratios also showed considerable variation, which again was greatest at low copy number. Moreover, when taking mean ratio values, our data shows that while integrated HPV16 in the absence of episomes may produce a low E2/E6 ratio, the presence of a high E2/E6 ratio, even one greater than 1.0, does not exclude the presence of integrated HPV16 only.

### Estimation of viral load and physical state of HPV16 in cervical clinical samples

The SYBR green qPCR method was also used to assess copy number of HPV16 genes in frozen tissue samples from fourteen HPV16-positive cervical SCCs (Table 5). Viral loads were determined as E6 copy number per diploid genome, and could broadly be classified into three levels; low (up to 50 viral copies per diploid genome); medium (approximately 300 to 700 viral copies per diploid genome); and high (above 2000 viral copies per diploid genome, seen in one sample only).

**Table 5:** NA6 qPCR analysis of E2 and E6 copy numbers in cervical SCC samples.

**Cervical SCC sample ID**	**E2 copies**	**E6 copies**	**Viral load level**	**Ratio of E2/E6**
**G30**	2	1	Low	1.28
**G1**	0	2	Low	0.21
**G31**	0	4	Low	0.06*
**n55**	0	5	Low	0.01*
**G19**	12	10	Low	1.26
**G12**	35	35	Low	0.99
**n12**	44	42	Low	1.03
**n10**	73	48	Low	1.54

**G26**	461	297	Medium	1.55
**G18**	258	343	Medium	0.75
**G6**	467	423	Medium	1.10
**G3**	384	528	Medium	0.73
**G9**	0	691	Medium	0.00*

**G11**	954	2,481	High	0.38

HPV gene copy numbers are per diploid genome. E2/E6 ratio values were calculated from the qPCR readings for E2 and E6 and rounded to the nearest two decimal places. The only samples in which HPV16 physical state could be inferred with confidence are marked by asterisks in the right hand column.

E2/E6 ratios were also determined for the SCCs. In three samples (n55, G9, G31), E2/E6 ratios were 0.06 or less, indicative of fully integrated HPV16 only (Table 5, '*' marked samples). In a fourth sample (G1) the ratio was 0.21, while in ten other samples it ranged from 0.38–1.55 (Table 5, right hand column). Such values have previously been considered to indicate the presence of episomes, with or without coexistence of integrants [5,11,24]. However, in view of our findings with cell lines, we concluded that these values may also represent integrated HPV16 only and that, consequently, no information regarding HPV16 physical state could be drawn with confidence from the E2/E6 values for these eleven samples.

## Discussion

HPV load and physical status have been claimed to be useful parameters for clinical evaluation of cervical squamous cell neoplasia [7,25-29]. Various PCR methodologies are described for estimation of HPV load, type and physical state [8,10,30-35]. Most of these techniques rely on the use of cloned full length HPV16 in order to produce a calibration curve for absolute quantification, with viral loads generally presented in terms of copies per unit mass of gDNA. However, thorough critical testing of these methods, using approaches similar to those in the present study, has been reported only rarely, and not at all for SYBR green based strategies.

Quantification of E2 and E6 relative to gDNA mass is subject to sources of potential error, including the need to quantify gDNA by spectrophotometry. Indeed it appears to have generated an error in the assessment of viral load in SiHa, which was determined in one study to be 172,991 copies of E6 in 50 ng of gDNA [13]. 50 ng of gDNA is equivalent to 4,630 triploid cells, giving a viral load in SiHa of 37 copies per cell, a substantial overestimate of the copy number demonstrated by Southern blotting. Such error may be reduced by using a calibration curve generated from a single clone, such as NA6, containing E2, E6 and HMBS amplicons in a 1:1:1 ratio. The NA6 standard curve enables the number of diploid genomes in a sample to be determined from the HMBS copy number. Although we have not performed direct comparisons, this approach should offer some advantages over alternative standardisation methods, as it negates the need to determine each sample concentration accurately, or to have knowledge of ploidy, and provides a reference point for comparison of unrelated specimens and PCR runs.

We undertook rigorous testing of the performance of SYBR green absolute qPCR in determining HR-HPV gene copy number. In experiments using virus host DNA mixtures we observed substantial run to run variation in PCR data and identified potential sources of error in copy number determination. Firstly, we observed that using an individual calibration curve produced variation in gene quantification of up to 31%, emphasizing the need to derive a standard curve from multiple replicate qPCR runs. Four such runs were used in our study.

Secondly, when we investigated the ability of SYBR green qPCR to derive gene copy numbers from test gDNA in which known amounts of HPV16 DNA had been added, we found moderate variation in quantification of individual E2 and E6 amplicons. This is consistent with the inherent potential for error in the procedures that we undertook in the relevant experiments, including estimating DNA concentration by spectrophotometry and serially diluting test samples. Estimates of viral load that were internally normalised to HMBS copy number showed error rates of 13–53%, a limitation that should be kept in mind when performing qPCR work.

In contrast to other studies in the literature, we also critically evaluated the performance of our SYBR green qPCR method in quantifying HPV16 gene copy number in a range of cervical keratinocyte cell lines. We observed reasonable agreement between viral loads determined by qPCR and Southern blotting, with some overestimation of low viral copy number by qPCR. The SYBR green method that we used determined an HPV16 copy number in SiHa of 5 copies per cell, which is an order of magnitude more accurate than a study that used gDNA levels quantified by spectrophotometry [13].

Our cell line data therefore suggests that our estimates of viral load (ranging from 2 to 2,000 copies per diploid genome) in a set of fourteen HPV16 positive cervical SCC clinical samples are likely to be broadly accurate, albeit subject to the errors that we demonstrated using HPV16/gDNA mixtures. Taken together, our detailed evaluation supports the use of SYBR green HPV16 qPCR in studies attempting to correlate viral copy number with clinical outcome using tissue specimens, at least where the degree of sampling of abnormal tissue is known.

We observed a wide range of E2/E6 ratios in cell lines and clinical samples. In integrant only SiHa cells the E2/E6 ratio was substantially greater than 0, at 0.2 (0.11–0.24), while in integrant only CaSki cells it was 2.61 (1.37–3.46), as a result of multiple integrants with greater representation of E2 than E6. These values (and their ranges) are attributable to the errors implicit in HPV gene quantification by qPCR, as well as to the retention of E2 sequences in some integrants. The latter may be full-length E2, as in CaSki, or alternatively fragments of E2 retaining the hinge region that is amplified by the most frequently used gene quantification primers (including those in the present study). While the hinge is the region of E2 that is most commonly deleted in HPV16 integration, it may also be retained; as is the case in the integrant in W12.Ser1p57 [18]. It should be noted that an excess of E2 copy number over E6 copy number is likely to be rare in cervical SCCs and only encountered in cells with high level genomic instability, such as CaSki. On the other hand, W12.Ser1p57 appears to be more representative of cells in vivo, with a low copy number of integrated HPV16. In W12.Ser1p57 we observed a mean E2/E6 ratio of 0.72, with a range of 0.57 to 3.82.

The E2/E6 ratios in episome-containing W12Ser1p16 cells ranged from 0.76–1.32 in triplicate assays. Nagao et al observed E2/E6 ratios of 0.61–1.13 in cervical carcinomas that were confirmed to contain HPV16 episomes only [24]. This group also identified cases with mixed integrant and episome infection in which the E2/E6 ratio was 0.41–0.55, consistent with their findings from experiments mixing plasmids containing full length HPV16 DNA and HPV6 E6 DNA [24]. Our current data suggests that E2/E6 ratios in this range could also reflect integrated HPV16 only and the presence of episomes cannot be assumed in such cases.

In our opinion, only very low E2/E6 ratios are likely to be good indicators of the integrant only state. We propose an E2/E6 ratio of 0.10 or less, as values greater than this are unlikely to discriminate reliably between integrant only samples and samples where episomes are present. A previous study showed that E2/E6 ratios under 0.10 were only seen where integrated HPV16 DNA was in 10 fold or greater excess over episomal HPV16 DNA [5]. However even these findings are rather questionable, as the qPCR assay used (in which gene copy numbers were referenced to a fixed mass of gDNA) gave an E2/E6 ratio of 0.25 when integrated and episomal DNA were present in a 1:1 mixture and 0.56 when episomal DNA was in 10 fold excess. Moreover, as with most published reports, no absolute copy number values were reported in the study [5].

E2/E6 ratios less than 0.10 are likely to be encountered rarely. Indeed, in our study we observed such values in only three of 14 HPV16 positive cervical SCCs. In the other cases, we consider that no reliable conclusions regarding viral state could be drawn from the E2/E6 ratio, indicating its limited usefulness when applied to clinical samples and uncharacterized cell lines. Based on these findings, we suggest that data from previous studies using the E2/E6 ratio to examine the physical state of HPV16 in clinical samples may not be accurate, and, in particular may have overestimated the frequency of mixed integrant and episomal infections in cervical neoplasia [5,13].

## Authors' contributions

IR devised the experiments, prepared the NA6 calibrator clone, and carried out accuracy tests and assessment of cell lines by qPCR. GN undertook assessment of clinical samples by qPCR and NF prepared calibration curves. W12 cell line establishment, propagation and gDNA preparations were made by MS and MRP. MTH prepared other cell line gDNA and contributed to the development of the qPCR method. AT provided statistical input and advised on qPCR mathematics. IR and NC wrote the manuscript. This work was funded by MRC and CRUK programme grants held by NC.

## Supplementary Material

Additional file 1 Crossing point values used in critical evaluation of HPV16 gene copy number quantification by SYBR green PCR. The Microsoft Excel workbook of Additional file 1 contains four worksheets. **1: NA6 Standard Curves.** The crossing point values used in generation of external calibration curves for absolute quantification of viral E2, viral E6 and host HMBS genes are presented. For each gene, four runs of a seven point NA6 titration series were conducted in triplicate. **2: Accuracy Test.** The crossing point values used to determine viral load and physical state of a three point serial dilution of a mixture of test gDNA and HPV16 plasmid DNA are presented. Three runs were undertaken at each titration point in triplicate. **3: Assessment of Cell Lines.** The crossing point values used to derive viral load and physical state of five cervical carcinoma cell lines are presented. Each cell line was assessed in three separate runs, and each reaction was performed in triplicate. **4: Assessment of Clinical Samples.** The crossing point values used to derive viral load and physical state of 14 squamous cell cervical carcinoma samples are presented. One run was undertaken for each sample, and all reactions were performed in triplicate. Click here for file
